# A novel genotype of “*Anaplasma capra*” in wildlife and its phylogenetic relationship with the human genotypes

**DOI:** 10.1038/s41426-018-0212-0

**Published:** 2018-12-12

**Authors:** Jifei Yang, Zhijie Liu, Qingli Niu, Muhammad Uzair Mukhtar, Guiquan Guan, Guangyuan Liu, Jianxun Luo, Hong Yin

**Affiliations:** 1 State Key Laboratory of Veterinary Etiological Biology, Key Laboratory of Veterinary Parasitology of Gansu Province, Lanzhou Veterinary Research Institute, Chinese Academy of Agricultural Sciences, Xujiaping 1, Lanzhou, Gansu 730046 China; 2 Jiangsu Co-innovation Center for Prevention and Control of Important Animal Infectious Diseases and Zoonoses, Yangzhou, 225009 China

*Anaplasm*a spp. are gram-negative obligate intracellular bacteria of remarkable importance in both human and veterinary health. Six *Anaplasma* species have currently been classified in the genus *Anaplasma* and are known as causative agents of tick-borne diseases^1^. The impacts of the diseases caused by *Anaplasma* on the productivity and health of animals have been recognized for over a century, and it is still a significant threat to livestock industries^2^. A zoonotic role has recently been uncovered for *Anaplasma phagocytophilum*, resulting in a rising interest in these organisms^3^. *Anaplasma phagocytophilum* has been considered the main cause of human anaplasmosis worldwide in recent years. Recently, a potential novel tick-transmitted *Anaplasma* species was identified in asymptomatic goats in China, and it has been recognized as a cause of human infection; the name “*Anaplasma capra*” was proposed provisionally^4^. Sequence and phylogenetic analyses of “*Anaplasma capra*” based on different gene loci revealed that this organism was distinct from all known *Anaplasma* species^4^. Although “*A. capra*” has not yet been formally recognized, human cases of “*A. capra*” infection have been recorded in northeastern China, and subsequent reports have also shown that “*A. capra*” seems to be widely distributed in China^4–7^, suggesting this agent may pose a substantial public health concern and may be responsible for anaplasmosis cases of unknown cause.

Wild animals are important reservoirs for tick-borne pathogens. The zoonotic *A. phagocytophilum* has been identified in a great number of wild animal species^2^. In previous reports, specific DNA of “*A. capra*” has been detected in domestic and some species of wild animals^5,7–9^, and “*A. capra*”-like bacteria were identified in ticks^10^, indicating “*A. capra*” might have a broad host range and genetic diversity.

This study was conducted in wild animals from Tangjiahe National Nature Reserve, which is a national protected area located in Sichuan Province, China (Supplementary Figure S1). This reserve is one of the biodiversity hotspots in the world and is an important natural habitat for a great number of endangered wildlife. From March 2016 to April 2017, 13 dead free-ranging wild animals were found in the field, including the first-grade nationally protected takin (*Budorcas taxicolor*) (five) and forest musk deer (*Moschus berezovskii*) (one); the second-grade nationally protected Himalayan goral (*Naemorhedus goral*) (three); and two nonendangered protected species, Reeves’s muntjac (*Muntiacus reevesi*) (three), and wild boar (one). Blood or liver samples were collected individually from animals, frozen at −20 °C, and transported in a freezer pack to a laboratory immediately. The total DNA was extracted using a QIAamp DNA Mini Kit according to the protocol. This study was approved by the Animal Ethics Committee of Lanzhou Veterinary Research Institute, Chinese Academy of Agricultural Sciences.

AllDNA samples were screened for the presence of “*A. capra*” by nested PCR targeting on *gltA* gene as previously described^4,10^. Positive (DNA extracted from a goat infected by “*A. capra*”, KX417336) and negative controls (double-distilled water) were included in each assay. “*Anaplasma capra*” was detected in six wild animals, including three takins, two Reeves’s muntjacs, and one forest musk deer (Supplementary Table S1). To further characterize the “*A. capra*” strains identified in this study, the 16S rRNA and *msp4* genes were amplified from the *gltA* gene-positive samples^5,10^. The PCR products of the partial 16S rRNA (1261 bp), *gltA*(594 bp), and *msp4*(656 bp) gene were purified using the AxyPrep DNA gel extraction kit (Axygen, USA), cloned (pGEM-T Easy vector, Promega, USA) and sequenced (GenScript, Nanjing, China). Sequence analysis revealed that the 16S rRNA sequences (MH188286) obtained in this study were 100% identical to each other and to those of “*A. capra*” isolates identified from sheep (MF066918), *H. qinghaiensis* ticks (KX673825) and Japanese serows (AB509223); and they were 99.8% identical to those of the isolates identified from humans (HLJ-14, KM206273) and *H. longicornis* ticks (KP314237). Phylogenetic analysis based on the 16S rRNA gene demonstrated that the isolates were clustered within the clade inside the“*A. capra*” but distinct from other, well-defined *Anaplasma* species (Fig. 1a).

**Fig. 1:**
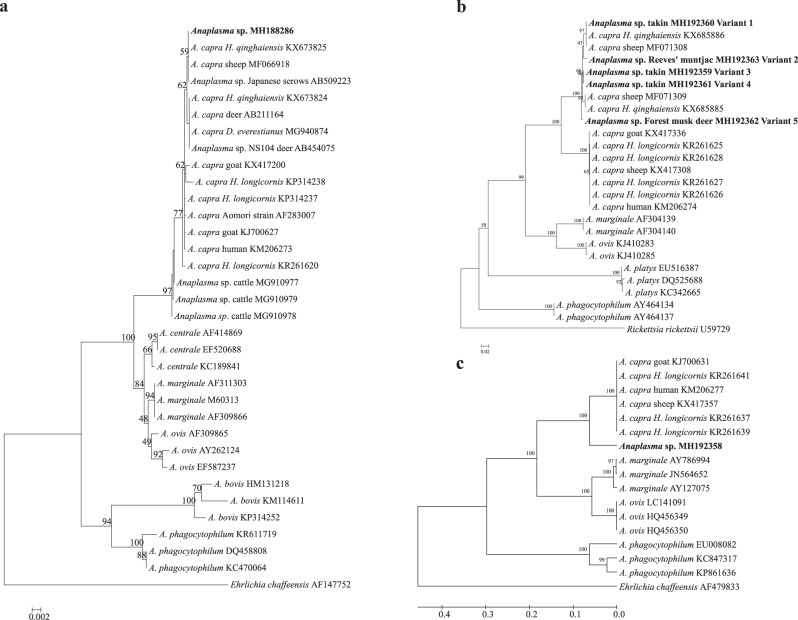
Phylogenetic analysis of “*Anaplasmacapra*” based on partial sequences of the 16S rRNA (1261 bp, **a**), *gltA* (594 bp, **b**), and *msp4* genes (656 bp, **c**). Phylogenetic trees were constructed based on the sequence distance method using the neighbor-joining (NJ) algorithm with the Kimura two-parameter model in MEGA 4.0 software. Bootstrap analysis was performed with 1000 replicates. *Ehrlichia chaffeensis* or *Rickettsia rickettsii* was used as the outgroup. Boldface indicates the sequences obtained in this study

According to the *gltA* sequence alignment, five sequence variants (MH192359–MH192363) with 2–6 bp nucleotide substitutions were obtained and showed 98.4–100% identity to those of “*A. capra*” isolates from sheep (MF071308 and MF071309) and *H. qinghaiensis* ticks (KX685885 and KX685886), but they were 87.2–88.3% identical to the isolate HLJ-14 from human (KM206274). Moreover, the *msp4*sequences of these isolates showed 88.4% identity to “*A. capra*” isolate HLJ-14 (KM206277). Phylogenetic analysis of both *gltA* and *msp4* gene sequences revealed that the “*A. capra*” strains were clustered independently from *Anaplasma* species identified previously and formed two distinct subclades with high bootstrap values. The isolates identified in this study were closely related to “*A. capra*” isolates from *H. qinghaiensis* and sheep but clearly distinct from human isolate HLJ-14 (Fig. 1b, c).

*Anaplasma* circulation in nature involves tick vectors and a great number of vertebrates that act as hosts and sources of infection for ticks, animals, and humans^2^. Since *Anaplasma* spp. are transmitted by ixodid ticks transstadially rather than transovarially, the reservoir hosts play a critical role in the maintenance and dispersal of these bacteria^11^. Numerous wild animal species have been considered competent reservoir hosts for *Anaplasma*, especially wild ruminants^11^, such as red deer (*Cervus elaphus*), roe deer (*Capreolus capreolus*), white-tailed deer(*Odocoileus virginianus*), mouflon (*Ovis musimon*), and chamois (*Rupicapra rupicapra*)^11^. In the present study, the novel tick-transmitted zoonotic “*A. capra*” was first identified in free-ranging wild animals (takin, Reeves’s muntjac, and forest musk deer) from a national nature reserve, where contact between wild and domestic animals rarely occurs. These results suggest that “*A. capra*” is endemic in this protected area and that those wild animals can act as competent sylvatic reservoirs for this organism.

The 16S rRNA gene sequence of “*A. capra*” was first described in cattle from Japan in 2001^12^, and the agent was mistakenly assumed to be *A. centrale* (Aomori strain, AF283007) (Fig. 1a)^13^. More recently, this novel *Anaplasma* species was recorded in goats from northeastern China, and it was subsequently identified as a human pathogen in an active surveillance of patients who sought treatment after a tick bite^4^. “*Anaplasma capra*” has been detected in several tick species, including *Ixodes persulcatus*, *Haemaphysalis longicornis*, and *Haemaphysalis qinghaiensis*^4,6,10^; however, the vector competence of these ticks for transmission of “*A. capra*” is still unclear and needs to be further evaluated. The infection of “*A. capra*” has also been reported in sheep and goats from several provinces of China^5^, in deer (*Anaplasma* sp. NS104, AB454075) and free-living serows (*Anaplasma* sp. Kamoshika17, AB509223) from Japan^8^, and in cattle (MG910977–9) from Malaysia^9^. Those findings together with the results obtained in this study suggested that additional tick species and/or domestic and wild animals might be involved in the transmission and maintenance of “*A. capra*”, which warrants further investigation.

Sequence and phylogenetic analysis based on the 16S rRNA gene demonstrated that “*A. capra*” isolates obtained from ticks, domestic, and wild animals as well as humans exhibit high sequence similarities to each other (99.8–100%) and cluster in a separate clade within the genus *Anaplasma*, but they are clearly distinct from the other members of *Anaplasma*, indicating the novelty of this causative agent. Obviously, these isolates of “*A. capra*” were a single species according to the classification criteria of bacteria^1,13^ (>99% 16S rRNA gene sequence similarity). However, they were classified into two different groups with low sequence similarity and a divergent phylogenetic relationship based on the *gltA* and *msp4* genes (87.2–88.3% for *gltA* and 88.4% for *msp4*), indicating two genotypes of “*A. capra*” in ticks and reservoir hosts. The “*A. capra*” genotype 1 identified in *I. persulcatus*, *H. longicornis*, sheep, goat, and human significantly differs from the genotype 2 identified in *H. qinghaiensis*, takin, Reeves’s muntjac and forest musk deer, suggesting a high degree of genetic diversity and host tropisms of “*A. capra*”, as has been documented in *A. phagocytophilum*^2^. However, it is unclear whether these two genotypes of “*A. capra*” have pathogenicity variation for animals and human, which should be clarified in the future.

In the past three decades, an increasing number of novel tick-associated microbes with zoonotic potential continue to be discovered, representing a considerable impact on public health worldwide. Since *A. phagocytophilum* was recognized as the causative agent of human granulocytic anaplasmosis, the cases have constantly increased in many countries^14^. However, numerous anaplasmosis cases of undetermined cause remain, indicating additional *Anaplasma* species are responsible for human anaplasmosis^2,15^. However, clinical cases of anaplasmosis might be generally neglected because the symptoms of the illness are notoriously nonspecific, making it likely to be confused with other illnesses^14^. The identification of the novel tick-transmitted zoonotic “*A. capra*” calls for clinicians to be aware of the possible infection from this causative agent in patients suspected of anaplasmosis or other tick-borne diseases, especially in areas where anaplasmosis can occur.

In summary, “*A. capra*” infection was first documented in three species of free-ranging wild ruminants from a national nature reserve of China, and a novel genotype of “*A. capra*” was identified that clearly differed from the genotype identified from human. Further studies should be conducted to fully elucidate the host range, vector ticks, pathogenicity, and geographic distribution of this organism.

## Supplementary Information

Supplementary Figure S1

Supplementary Table S1
