# Supplementary material for: A novel genotype of “Anaplasma capra” in wildlife and its phylogenetic relationship with the human genotypes
Source: Emerg Microbes Infect. 2018 Dec 12;7:210. doi: 10.1038/s41426-018-0212-0 (PMC6290010; doi:10.1038/s41426-018-0212-0)
Supplement: Supplementary file 1 — Supplementary Figure S1 [file 41426_2018_212_MOESM1_ESM.pdf]

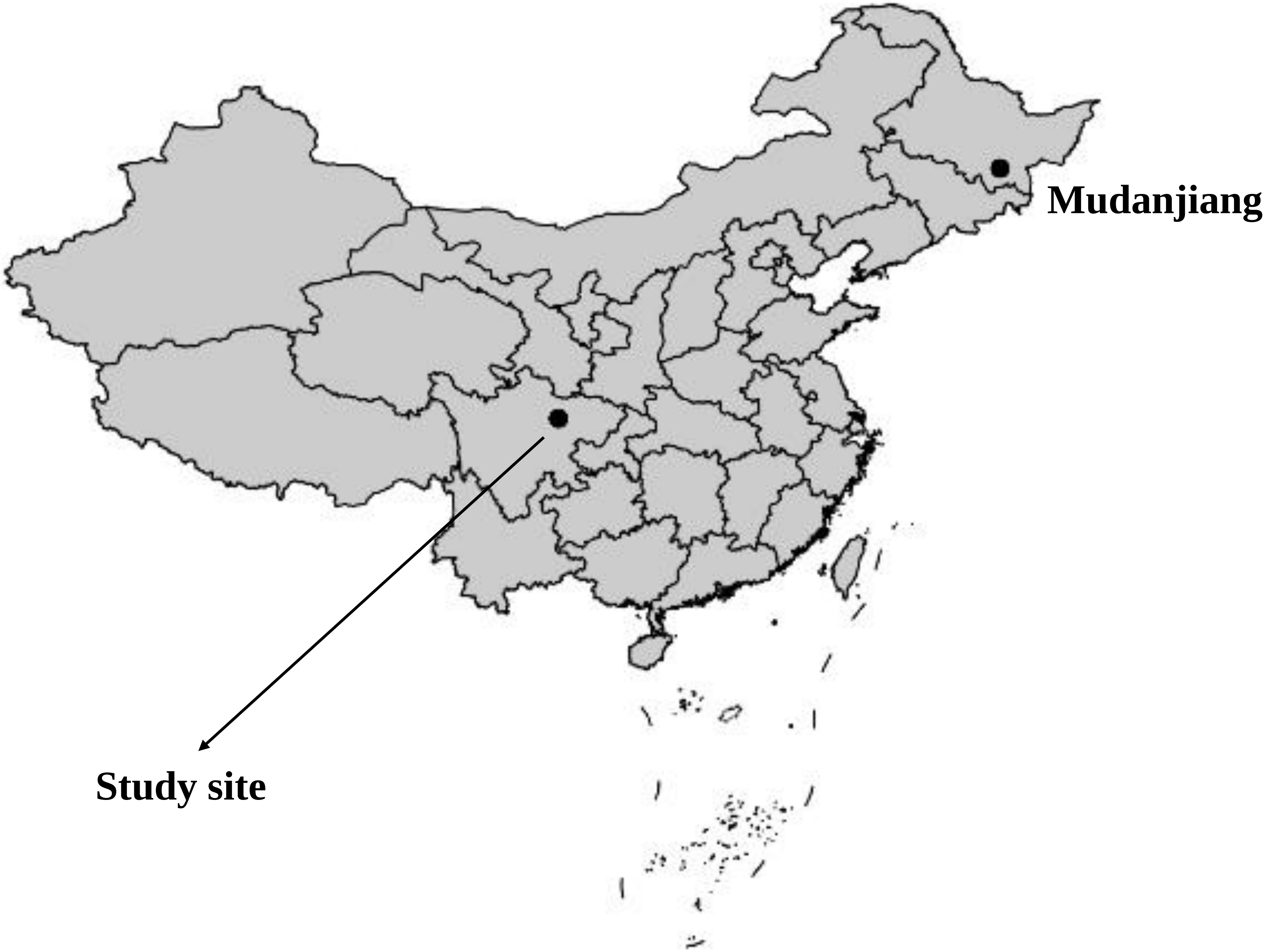

Figure S1. Study site in Tangjiahe National Nature Reserve of Sichuan Province of China. Black solid circle indicates the location (Mudanjiang) where "*Anaplasma capra*" was previously isolated. The study site for the current study is also indicated.
