# Supplementary material for: A novel genotype of “Anaplasma capra” in wildlife and its phylogenetic relationship with the human genotypes
Source: Emerg Microbes Infect. 2018 Dec 12;7:210. doi: 10.1038/s41426-018-0212-0 (PMC6290010; doi:10.1038/s41426-018-0212-0)
Supplement: Supplementary file 2 — Supplementary Table S1 [file 41426_2018_212_MOESM2_ESM.docx]

Table S1. “*Anaplasma* *capra*” variants identified in wildlife from Tangjiahe National Nature Reserve, China.

| Animal species | Scientific name | Sample | *gltA* sequence variants | GenBank accession numbers |
| --- | --- | --- | --- | --- |
| Himalayan goral | *Naemorhedus goral* | Blood | – | – |
| Himalayan goral | *Naemorhedus goral* | Liver | – | – |
| Himalayan goral | *Naemorhedus goral* | Liver | – | – |
| Takin | *Budorcas taxicolor* | Blood | Variant 3 | MH192359 |
| Takin | *Budorcas taxicolor* | Blood | – |  |
| Takin | *Budorcas taxicolor* | Blood | Variant 1 | MH192360 |
| Takin | *Budorcas taxicolor* | Liver | Variant 4 | MH192361 |
| Takin | *Budorcas taxicolor* | Liver | – |  |
| Reeves' muntjac | *Muntiacus reevesi* | Blood | – |  |
| Reeves' muntjac | *Muntiacus reevesi* | Blood | Variant 2 | MH192363 |
| Reeves' muntjac | *Muntiacus reevesi* | Liver | Variant 2 | MH192363 |
| Forest musk deer | *Moschus berezovskii* | Blood | Variant 5 | MH192362 |
| Wild boar | *Sus scrofa* | Liver | – |  |
